# Endothelial cells act as gatekeepers for LTβR-dependent thymocyte emigration

**DOI:** 10.1084/jem.20181345

**Published:** 2018-12-03

**Authors:** Kieran D. James, Emilie J. Cosway, Beth Lucas, Andrea J. White, Sonia M. Parnell, Manuela Carvalho-Gaspar, Alexei V. Tumanov, Graham Anderson, William E. Jenkinson

**Affiliations:** 1 Institute of Immunology and Immunotherapy, College of Medical and Dental Sciences, Medical School, University of Birmingham, Birmingham, UK; 2 Department of Microbiology, Immunology and Molecular Genetics, University of Texas School of Medicine, University of Texas Health Science Center, San Antonio, TX

## Abstract

The emigration of mature thymocytes from the thymus is critical for establishing peripheral T cell compartments. However, the pathways controlling this process and the timing of egress in relation to postselection developmental stages are poorly defined. Here, we reexamine thymocyte egress and test current and opposing models in relation to the requirement for LTβR, a regulator of thymic microenvironments and thymocyte emigration. Using cell-specific gene targeting, we show that the requirement for LTβR in thymocyte egress is distinct from its control of thymic epithelium and instead maps to expression by endothelial cells. By separating emigration into sequential phases of perivascular space (PVS) entry and transendothelial migration, we reveal a developmentally ordered program of egress where LTβR operates to rate limit access to the PVS. Collectively, we show the process of thymic emigration ensures only the most mature thymocytes leave the thymus and demonstrate a role for LTβR in the initiation of thymus emigration that segregates from its control of medulla organization.

## Abstract (precis)

Thymic emigration is essential for establishing T cell immunity. We show the requirement for LTβR segregates from its control of medullary epithelium. Instead, our study demonstrates LTβR expression by the endothelium acts to rate limit thymocyte egress via perivascular routes.

## Graphical Abstract

**Figure:**
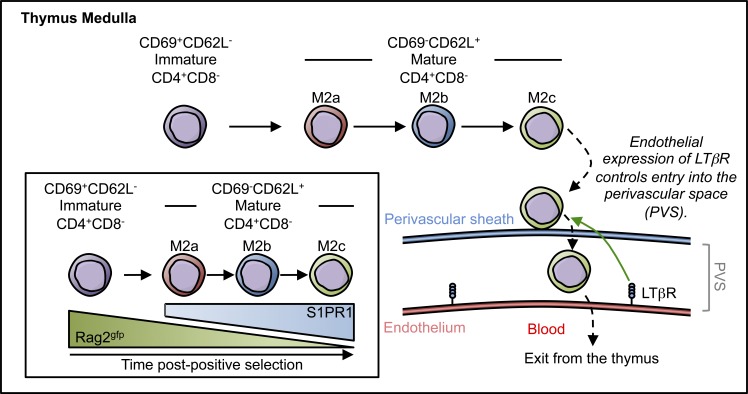

## Introduction

In the thymus, immature thymocytes with self-MHC restricted αβ TCRs undergo positive selection and relocate from the cortex to the medulla as single-positive (SP) CD4 (SP4) or CD8 (SP8) thymocytes in response to CCL21 production by medullary thymic epithelial cells (mTECs; Kozai et al., 2017). The medulla has multiple functions, including T cell tolerance via clonal deletion and regulatory T cell development (Akiyama et al., 2005; Cowan et al., 2013; Kozai et al., 2017). It also supports postselection maturation of conventional thymocytes, with transition through sequential SP stages involving NF-κB and type I interferon–dependent pathways that control TCR-mediated proliferation competency and cytokine licensing (Xing et al., 2016). Further, in the medulla, SP thymocytes acquire an exit phenotype, including expression of the transcription factor KLF2 and the cell surface receptor S1P_1_ that is essential for thymus emigration at the corticomedullary junction (CMJ; Matloubian et al., 2004; Zachariah and Cyster, 2010). Thus, to ensure the thymus exports newly formed T cells as recent thymic emigrants (RTEs) with appropriate functional capacities, the medulla must carefully coordinate phases of postselection thymocyte maturation with the process of egress.

Despite its significance, thymic emigration is poorly understood. First, the timing of thymic egress in relation to thymocyte age remains controversial. Thus, alternative models of either a random “lucky dip” egress of SP thymocytes at multiple maturational stages or synchronous egress of the oldest thymocytes via a “conveyor belt” mechanism remain to be fully tested (Scollay and Godfrey, 1995). Second, few pathways that control thymic egress are known. In this context, mice lacking lymphotoxin-β receptor (LTβR) show increased mature SP thymocytes (Boehm et al., 2003), which correlates with medullary disorganization and impaired mTEC development (Boehm et al., 2003; Venanzi et al., 2007; Martins et al., 2008). Collectively, these findings support a model where LTβR controls thymus emigration by its influence on thymic epithelium. However, as LTβR is expressed by both TEC and non-TEC stroma (Seach et al., 2008; Cosway et al., 2017), the cellular context and mechanism by which this key regulator influences thymic emigration is unknown.

Here, we investigate thymus emigration and reevaluate the role of LTβR in this process. By deletion of LTβR in specific thymic stroma compartments, we show that absence of LTβR expression by TECs disrupts medulla formation, but not thymic egress. Rather, LTβR regulates thymocyte emigration via expression on endothelial cells, where it controls access to perivascular portals for thymic egress. We also identify a conveyor belt mechanism of thymocyte emigration that is established independently of LTβR and demonstrate a role for LTβR in determining the rate of conveyor belt exit. Overall, we define LTβR-independent and -dependent mechanisms of thymic export, where endothelial LTβR expression controls the rate of ordered thymocyte egress via the perivascular route.

## Results and discussion

### LTβR controls intrathymic dwell time and egress of SP4 thymocytes

**Figure 1.**
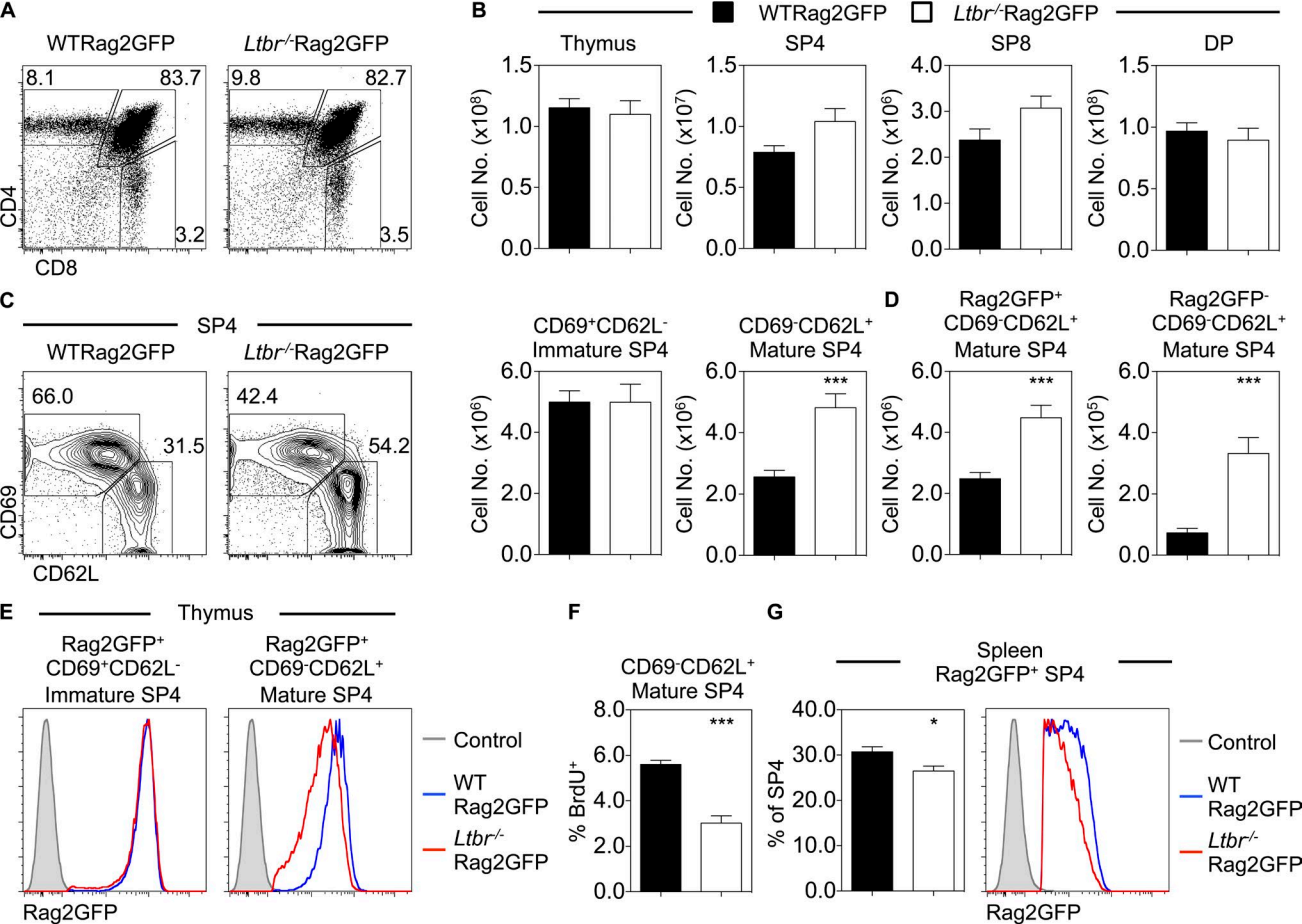
**LTβR regulates thymocyte egress. (A)** Thymocyte development in WTRag2GFP and *Ltbr*^−/−^Rag2GFP adult mice. **(B)** Numbers of total thymocytes, CD4^+^CD8^−^TCRβ^HI^CD25^−^ SP4, CD4^−^CD8^+^TCRβ^+^ SP8, and CD4^+^CD8^+^ double-positive (DP) thymocytes in WTRag2GFP (black bars, *n* = 9) and *Ltbr*^−/−^Rag2GFP adult mice (white bars, *n* = 12). Data are pooled from five independent experiments. **(C)** Analysis of CD69^+^CD62L^−^ (immature) and CD69^−^CD62L^+^ (mature) subsets gated on CD4^+^CD8^−^TCRβ^HI^CD25^−^ SP4. **(D)** Numbers of Rag2GFP^+^ and Rag2GFP^−^ mature SP4. **(E)** Rag2GFP levels in GFP^+^ immature (left) and mature (right) SP4, where gray filled histograms represent nonfluorescent WT staining control. **(F)** Percentage of BrdU^+^ mature SP4 thymocytes (*n* ≥ 5 pooled from two independent experiments). **(G)** Percentage of Rag2GFP^+^ CD4^+^TCRβ^HI^CD25^−^ splenic RTEs and their level of GFP expression in WTRag2GFP (black bars, blue line *n* = 9) and *Ltbr*^−/−^Rag2GFP adult mice (white bars, red line, *n* = 12). Data are pooled from five independent experiments, where the gray filled histogram indicates a nonfluorescent WT staining control. An unpaired Student’s *t* test was used for statistical analysis. All bar charts and error bars represent means ± SEM. *, P < 0.05; ***, P < 0.001.

Germline *Ltbr*^−/−^ mice display multiple thymic defects, including abnormal mTEC development and organization, reduced lymphoid progenitor colonization, and failure of central tolerance (Lucas et al., 2016; Shi et al., 2016; Cosway et al., 2017). Further, increased SP thymocytes in *Ltbr*^−/−^ mice suggest a role for LTβR in thymic egress (Boehm et al., 2003). To investigate how LTβR controls egress, we crossed *Ltbr*^−/−^ mice with Rag2GFP mice where GFP levels are indicative of thymocyte age and thymus residency (Yu et al., 1999; Boursalian et al., 2004; McCaughtry et al., 2007). Total thymocytes, as well as CD4^+^CD8^+^ (double positive), SP4, and SP8 subset distribution, were equivalent in *Ltbr*^−/−^Rag2GFP and WTRag2GFP mice (Fig. 1, A and B). Subdivision of CD25^−^TCRβ^HI^ SP4 thymocytes into immature CD69^+^CD62L^−^ and mature CD69^−^CD62L^+^ subsets revealed a selective increase in the latter in *Ltbr*^−/−^Rag2GFP thymus (Fig. 1 C). Subdivision of SP4 using alternative markers, including CD69/MHC class I and CD24/CD62L (Mouri et al., 2014; Xing et al., 2016; White et al., 2017), showed a similar selective increase of mature SP4 thymocytes (Fig. S1). Gating on GFP^+^ cells to discriminate new thymocytes from recirculating T cells (McCaughtry et al., 2007; Cowan et al., 2016), we found increased GFP^+^ SP thymocytes in *Ltbr*^−/−^Rag2GFP mice (Fig. 1 D). We also saw increased GFP^−^ SP4 cells representing recirculating cells (Fig. 1 D). While the reasons for this are unknown, it may be due to limited secondary lymphoid tissue niches in *Ltbr*^−/−^ mice (Fütterer et al., 1998).

As Rag2GFP levels indicate time spent in the thymus (McCaughtry et al., 2007), we analyzed this in GFP^+^ SP4 thymocytes from WTRag2GFP and *Ltbr*^−/−^Rag2GFP. While GFP levels in immature CD69^+^CD62L^−^ SP4 were equivalent, GFP levels in mature CD69^−^CD62L^+^ cells from *Ltbr*^−/−^Rag2GFP mice were reduced compared with WT mice (Fig. 1 E), suggesting that increased thymic dwell time explains the accumulation of mature SP4. Importantly, we saw no increase in BrdU^+^CD69^−^CD62L^+^ SP4 thymocytes in *Ltbr*^−/−^ mice; rather, BrdU^+^ cells were reduced (Fig. 1 F). While the reason for this is unclear, these data argue against enhanced dilution of GFP in *Ltbr*^−/−^ mice occurring due to increased proliferation. Analysis of splenic T cells in *Ltbr*^−/−^ mice revealed fewer Rag2GFP^+^ SP4 RTEs, with such cells displaying reduced GFP levels (Fig. 1 G). Collectively, these data show that SP4 alterations in *Ltbr*^−/−^ mice are a direct result of defective thymus emigration and explain the increased number of mature CD69^−^CD62L^+^ SP thymocytes as a consequence of prolonged thymic dwell time.

### LTβR expression by endothelial cells regulates thymic egress

**Figure 2.**
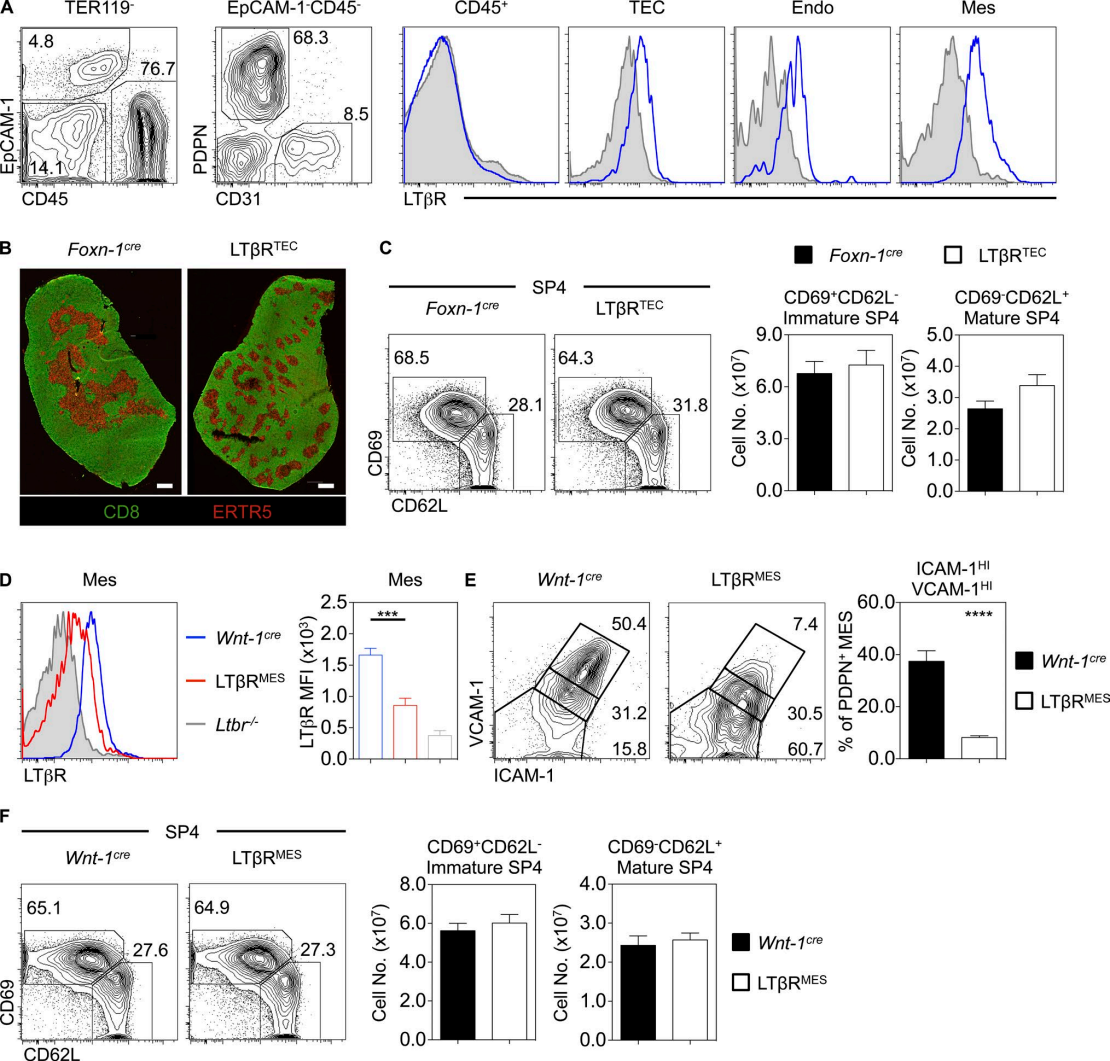
**LTβR on thymic epithelium is dispensable for thymus emigration. (A)** LTβR expression on TER119^−^CD45^+^ hematopoietic cells, TER119^−^CD45^−^EpCAM-1^+^ TECs, TER119^−^CD45^−^EpCAM-1^−^PDPN^+^CD31^−^ mesenchyme (Mes), or TER119^−^CD45^−^EpCAM-1^−^PDPN^−^CD31^+^ endothelium (Endo). Gray filled histograms represent control staining in *Ltbr^−/−^*. Data represent *n* = 4 from three independent experiments. **(B)** Thymus sections stained with anti-CD8 (green) and ERTR5 (red) to detect medullary epithelium in indicated strains. Scale bar: 500 µm. Images represent *n* > 4. **(C)** CD69^+^CD62L^−^ immature and CD69^−^CD62L^+^ mature CD4^+^CD8^−^TCRβ^HI^CD25^−^Foxp3^−^ SP4 in *Foxn-1^cre^* (black, *n* = 13) and LTβR^TEC^ mice (white, *n* = 14). Data are pooled from four independent experiments. **(D)** LTβR expression on TER119^−^CD45^−^EpCAM-1^−^PDPN^+^CD31^−^ thymic mesenchyme in *Wnt-1^cre^* (blue bars, *n* = 7) or LTβR^MES^ mice (red bars, *n* = 9) mice, where the gray filled histogram indicates control *Ltbr*^−/−^ staining. Data are pooled from two independent experiments. **(E)** Percentage of ICAM-1^HI^VCAM-1^HI^ thymic mesenchyme in *Wnt-1^cre^* (black bars, *n* = 6) and LTβR^MES^ mice (white bars, *n* = 6). Data are pooled from two independent experiments. **(F)** Analysis of immature and mature SP4 thymocytes in *Wnt-1^cre^* (black bars, *n* = 6) and LTβR^MES^ (white bars, *n* = 8) adult mice. Data are pooled from three independent experiments. An unpaired Student’s *t* test was used for statistical analysis. All bar charts and error bars represent means ± SEM. ***, P < 0.001; ****, P < 0.0001.

Given the widespread LTβR expression by multiple thymic stromal cell types (Fig. 2 A; Shi et al., 2016; Sitnik et al., 2016; Cosway et al., 2017), any cell type–specific requirements for LTβR during thymus emigration are unknown. To address this, we generated mice lacking LTβR in specific stromal cells. First, we analyzed *Foxn1^Cre^Ltbr^fl/fl^* (LTβR^TEC^) mice, where LTβR deletion occurs in TECs and leads to quantitative loss of mTECs, including the CCL21^+^ subset (Cosway et al., 2017). Consistent with cell-intrinsic regulation of TEC by LTβR, we saw disorganized medullas in LTβR^TEC^ mice, mirroring those of *Ltbr*^−/−^ mice (Fig. 2 B). Importantly, and in contrast to *Ltbr*^−/−^ mice, the numbers of mature CD69^−^CD62L^+^ SP4 thymocytes in LTβR^TEC^ mice were comparable to *Foxn1^Cre^* controls (Fig. 2 C). Thus, alterations in thymic egress in *Ltbr*^−/−^ mice do not map to defects in mTECs.

To see if thymic emigration is influenced by LTβR conditioning of mesenchyme, a key regulator of thymic egress (Zachariah and Cyster, 2010), we generated *Wnt1^Cre^Ltbr^fl/fl^* (LTβR^MES^) to delete LTβR from neural crest–derived mesenchyme (Fig. 2 D). Consistent with a role for LTβR in mesenchyme maturation (Sitnik et al., 2016), we saw loss of ICAM1^HI^VCAM1^HI^ mesenchyme in LTβR^MES^ mice (Fig. 2 E). Comparison of thymocyte development in LTβR^MES^ and *Wnt-1^Cre^* controls showed no impact on mature CD69^−^CD62L^+^ SP4 (Fig. 2 F). Thus, LTβR regulates thymic emigration independently of thymic mesenchyme.

**Figure 3.**
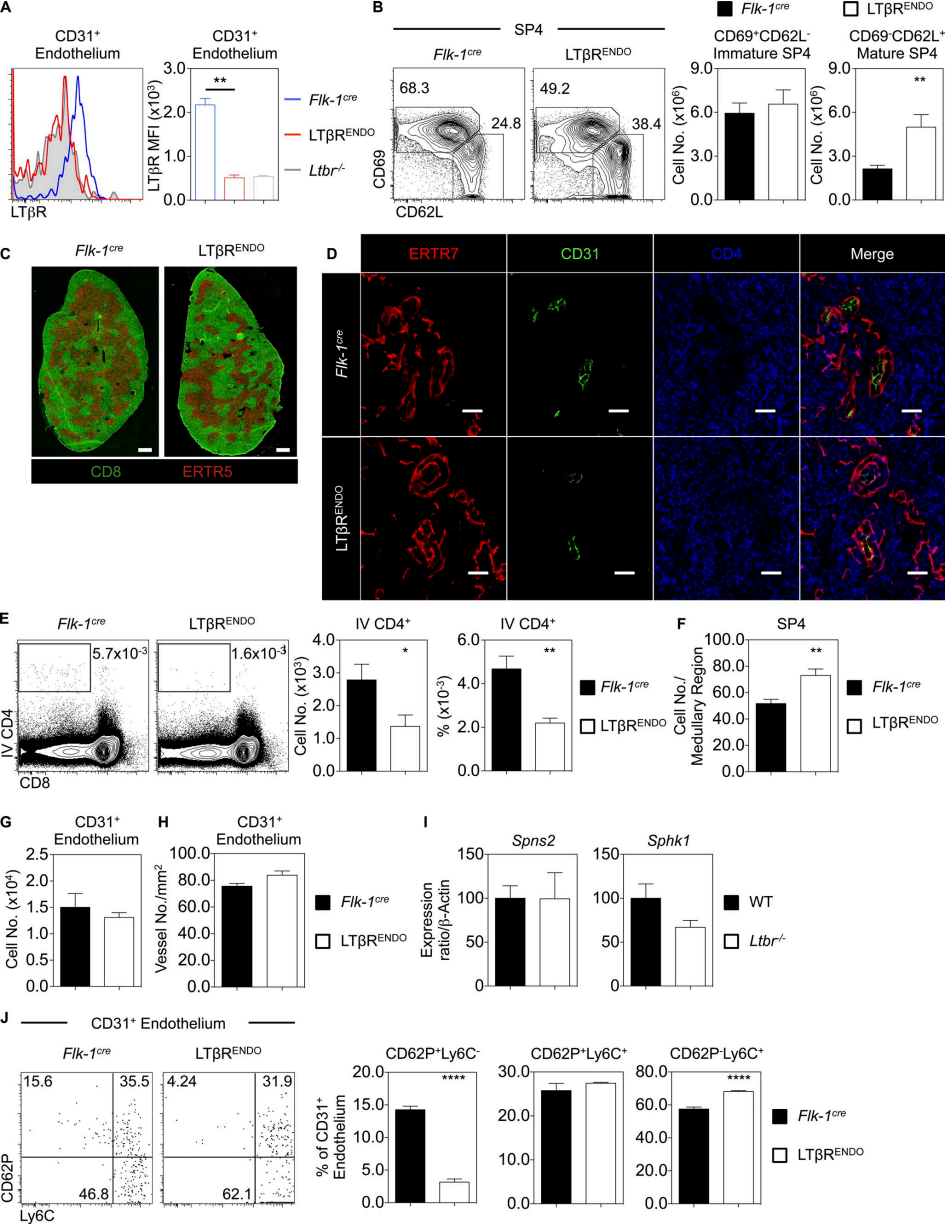
**LTβR expression on the endothelium regulates thymocyte egress via the perivascular route. (A)** Analysis of LTβR on TER119^−^CD45^−^EpCAM-1^−^PDPN^−^CD31^+^ thymic endothelium in *Flk-1^cre^* (blue, *n* = 6) and LTβR^ENDO^ (red, *n* = 5), where the gray filled histogram indicates staining in an *Ltbr*^−/−^ mouse. Data are pooled from two independent experiments. **(B)** CD69^+^CD62L^−^ immature and CD69^−^CD62L^+^ mature CD4^+^CD8^−^TCRβ^HI^CD25^−^Foxp3^−^ SP4 thymocytes in *Flk-1^cre^* (black, *n* = 9) and LTβR^ENDO^ (white, *n* = 7). Data are pooled from three independent experiments. **(C)** Thymus sections stained with anti-CD8 (green) and ERTR5 (red) in *Flk-1^cre^* (left) and LTβR^ENDO^ (right). Scale bars: 500 µm. Images represent *n* ≥ 4 mice. **(D)** Thymus sections from *Flk-1^cre^* (top) and LTβR^ENDO^ (bottom) stained for ERTR7 (red), CD31 (green), and CD4 (blue) to identify thymocytes around and in the PVS. Scale bars: 20 µm. Images represent *n* = 3 mice. **(E)** Percentage and number of i.v. anti-CD4 PE-labeled thymocytes in *Flk-1^cre^* (black, *n* = 6) and LTβR^ENDO^ mice (white, *n* = 6). Data are pooled from two independent experiments. **(F)** Quantitation of SP4 thymocytes in medullary regions of *Flk-1^cre^* (black bars) and LTβR^ENDO^ mice (white bars). Data represent mean number of cells counted per medullary area (*n* = 3 mice per strain). **(G)** Number of CD31^+^ thymic endothelial cells in *Flk-1^cre^* (black, *n* = 7) and LTβR^ENDO^ (white, *n* = 6). Data are pooled from two independent experiments. **(H)** Confocal quantitation of medullary ERTR7^+^ perivascular-associated CD31^+^ vessels located within 100 µm of the CMJ in *Flk-1^cre^* (black, *n* = 3) and LTβR^ENDO^ mice (white, *n* = 3). **(I)** qPCR of indicated genes in purified CD31^+^ WT and *Ltbr*^−^*^/^*^−^ thymic endothelium. Data are from three independent repeats. **(J)** CD62p and Ly6C expression on CD31^+^ thymic endothelium and percentage of CD62p^+^Ly6C^−^, CD62p^+^Ly6C^+^, and CD62p^−^Ly6C^+^ CD31^+^ thymic endothelium in *Flk-1^cre^* (black, *n* = 7) and LTβR^ENDO^ mice (*n* = 6). Data are pooled from two independent experiments. An unpaired Student’s *t* test was used for statistical analysis. All bar charts and error bars represent means ± SEM. *, P < 0.05; **, P < 0.001; ****, P < 0.0001.

Given that thymic egress involves migration across blood–endothelial barriers (Zachariah and Cyster, 2010) and robust LTβR expression by thymic endothelium (Fig. 2 A), we generated *Flk1^Cre^Ltbr^fl/fl^* (LTβR^ENDO^) mice to delete LTβR on endothelium (Fig. 3 A). Similar to *Ltbr*^−/−^ mice, we saw a significant and selective accumulation of mature CD69^−^CD62L^+^ SP4 thymocytes in LTβR^ENDO^ mice (Fig. 3 B). In contrast to *Ltbr*^−/−^ mice, confocal analysis revealed normal organization of medullary microenvironments in LTβR^ENDO^ thymus (Fig. 3 C). Thus, the role of LTβR in thymocyte emigration maps to expression by thymic endothelial cells and is separate from LTβR control of medulla topology.

### LTβR expression by endothelium controls perivascular space (PVS) entry

During thymic exit, SP thymocytes enter the PVS situated between the vascular basement membrane and encircling neural crest–derived mesenchyme (Zachariah and Cyster, 2010). Alterations in thymic emigration can manifest as SP accumulations in the PVS, which can be detected by confocal microscopy (Mori et al., 2007; Maeda et al., 2014; White et al., 2017). Given thymus egress defects in LTβR^ENDO^ mice, we examined the anatomical basis of SP4 thymocyte accumulation. Thymic sections from LTβR^ENDO^ and *Flk1^Cre^* controls were stained with anti-CD31 and ERTR7 (to identify endothelium and basement membranes) and anti-CD4 (to detect SP4 thymocytes in medulla areas). Confocal analysis of LTβR^ENDO^ mice revealed PVS structures lying between the ERTR7^+^ basement membrane and CD31^+^ endothelium (Fig. 3 D). In contrast to *Il4ra*^−/−^ mice, where altered thymic egress manifests as enlarged PVSs (White et al., 2017), we failed to detect PVS accumulations of SP4 in LTβR^ENDO^ mice (Fig. 3 D). To provide quantitative analysis, we used i.v. anti-CD4 PE antibody labeling to identify SP4 thymocytes in the PVS (Zachariah and Cyster, 2010). Strikingly, compared with control mice, LTβR^ENDO^ mice exhibited a significant reduction in PE-labeled SP4 thymocytes in the PVS (Fig. 3 E). Therefore, endothelial LTβR deficiency results in reduced PVS entry, suggesting the block in thymic egress occurs upstream of this stage. To examine this, we quantitated numbers of SP4 in thymic sections. LTβR^ENDO^ mice showed increased SP4 numbers in the medulla compared with control mice (Fig. 3 F). Together, these data demonstrate that LTβR controls T cell egress by regulating PVS entry.

We next sought to further examine how LTβR regulation of endothelium controls thymic egress. The number of CD31^+^ endothelial cells was normal in LTβR^ENDO^ mice (Fig. 3 G), as was the number of PVS-associated blood vessels in the CMJ, which represent the major points for thymic egress (Zachariah and Cyster, 2010; Fig. 3 H). Thus, impaired egress in LTβR^ENDO^ mice is not due to reduced availability of thymic exit portals. As S1P production by stroma, including endothelium, is critical for thymocyte egress (Pappu et al., 2007; Zachariah and Cyster, 2010; Fukuhara et al., 2012), we examined the expression of key molecules linked to this process. Expression of the sphingosine kinase *Sphk1* and the S1P transporter *Spns2* revealed no major reduction in endothelium isolated from LTβR^ENDO^ mice (Fig. 3 I), suggesting LTβR controls egress independently of these molecules. Finally, a novel LTβR-dependent Ly6C^−^CD62P^+^ thymic endothelial subset termed the portal endothelium has been recently identified and linked to regulation of lymphoid progenitor cell entry (Shi et al., 2016). In agreement with these studies, we observed significant loss of Ly6C^−^CD62P^+^ portal endothelium in LTβR^ENDO^ mice (Fig. 3 J). Together, these data demonstrate that LTβR controls the formation and/or maintenance of defined thymic endothelial subsets and raises the possibility that such cells may act to link thymus entry and exit.

### Thymic egress via the perivascular route occurs in a conveyor belt manner

**Figure 4.**
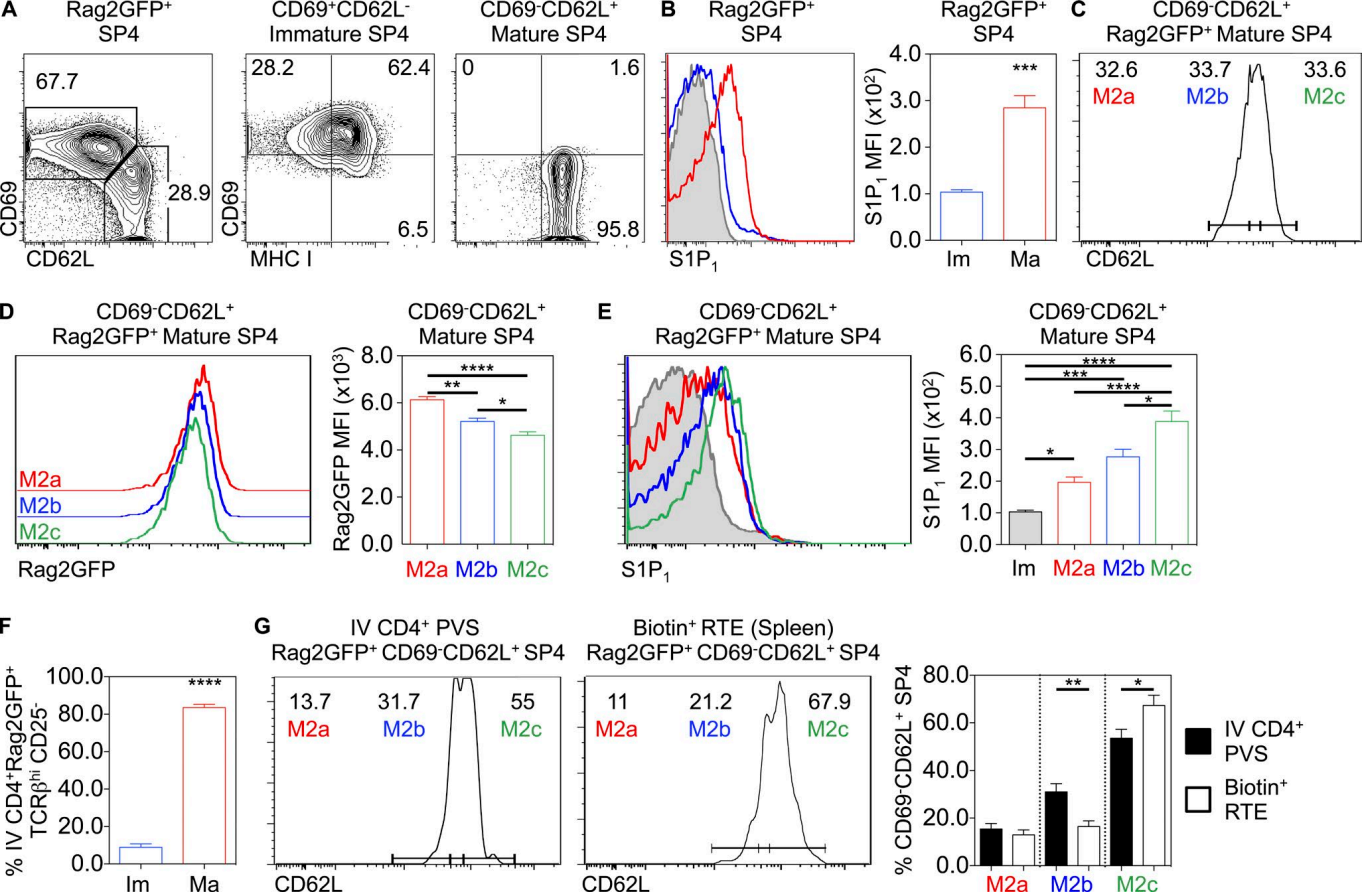
**A conveyor belt mechanism of thymocyte egress. (A)** CD69, MHC I expression on CD69^+^CD62L^−^ immature and CD69^−^CD62L^+^ mature CD4^+^CD8^−^TCRβ^HI^CD25^−^ Rag2GFP^+^ SP4. **(B)** S1P_1_ expression on immature (Im; blue, *n* = 5) and mature (Ma; red, *n* = 5) Rag2GFP^+^ SP4 thymocytes, where the gray filled histogram indicates fluorescence-minus-one staining control. Data are pooled from three independent experiments. **(C)** Mature CD69^−^CD62L^+^ Rag2GFP^+^ SP4 thymocytes separated into three equal populations based on CD62L, where CD62L^low^ = M2a, CD62L^int^ = M2b, and CD62L^high^ = M2c. **(D)** Rag2GFP expression in M2a, M2b, and M2c (*n* = 6). Data are pooled from three independent experiments. **(E)** S1P_1_ expression on M2a, M2b, and M2c subsets (*n* = 6), where the gray filled histogram represents immature SP4 thymocytes as a control. Data are pooled from three independent experiments. **(F)** Percentage of i.v. anti-CD4 PE-labeled PVS-associated immature (blue, *n* = 8) and mature (red, *n* = 8) SP4 in WTRag2GFP mice. **(G)** Percentage of M2a, M2b, and M2c subsets in i.v. anti-CD4 PE-labeled PVS-associated SP4 (left plots, black bars, *n* = 8) and splenic CD4^+^TCRβ^HI^CD25^−^ RTEs (right plot, white bars, *n* = 9). Data are pooled from three or more experiments. An unpaired Student’s *t* test (B and F) or one-way ANOVA (D, E, and G) was used for statistical analysis. All bar charts and error bars represent means ± SEM. *, P < 0.05; **, P < 0.01; ***, P < 0.001; ****, P < 0.0001.

Given conflicting results on conveyor belt or stochastic lucky dip mechanisms of thymic exit (Scollay and Godfrey, 1995; McCaughtry et al., 2007), we reevaluated the timing of thymic emigration in relation to our findings on the importance of LTβR. First, we looked for evidence of developmental heterogeneity in mature SP4 thymocytes shown previously to contain egress-competent cells (McCaughtry et al., 2007). We focused on CD69^−^CD62L^+^ SP4 thymocytes from WT Rag2GFP mice, which are exclusive to the most mature CD69^−^MHCI^+^ M2 subset (Fig. 4 A; Xing et al., 2016) and, unlike their immature progenitors, express S1P_1_ (Fig. 4 B; Allende et al., 2004; Matloubian et al., 2004). We separated CD69^−^CD62L^+^ SP4 thymocytes into three equal subsets based on increasing levels of CD62L, a downstream target of the transcription factor KLF2 that controls thymic exit (Carlson et al., 2006), and designated them as M2a (CD62L^low^), M2b (CD62L^int^), and M2c (CD62L^high^; Fig. 4 C). Consistent with maturational heterogeneity within total CD69^−^CD62L^+^ cells, GFP levels were highest in M2a cells, followed by M2b cells and then M2c cells (Fig. 4 D). Compared with immature CD69^+^CD62L^−^ SP4 thymocytes, M2a, M2b, and M2c SP4 thymocytes all expressed S1P_1_ (Fig. 4 E), with the highest levels detected on the most mature M2c cells (Fig. 4, D and E). These findings indicate a temporal sequence of M2a-M2b-M2c in CD69^−^CD62L^+^ SP4 thymocytes and suggest that transition through these stages involves progressive up-regulation of the egress regulator S1P_1_.

Our finding that developmental heterogeneity occurs within mature CD69^−^CD62L^+^ SP4 is relevant to proposed models of thymic exit. In SP4 cells undergoing thymic egress, a conveyor belt mechanism of exit would lead to selective enrichment of the most mature S1P_1_^high^ M2c cells in the PVS. Conversely, equal distribution of M2a/M2b/M2c cells in the PVS may point toward a stochastic mechanism of exit. To distinguish between these possibilities, we labeled cells in the PVS using i.v. anti-CD4 PE. Consistent with their selective expression of S1P_1_, almost all PVS-residing SP4 thymocytes were mature CD69^−^CD62L^+^ cells (Fig. 4 F). When we analyzed CD62L levels in PVS-labeled cells, we saw a bias toward the most mature M2c cells (Fig. 4 G), with fewer M2a and M2b cells. Thus, CD69^−^CD62L^+^ SP4 thymocytes in the PVS are enriched in the most mature M2c subset. To determine whether ordered entry of M2c cells into the PVS correlates with a preferential ability to exit the thymus and enter the periphery, we assessed the developmental status of RTEs using intrathymic labeling. Adult WT mice were intrathymically injected with biotin, and biotin^+^ RTEs in the spleen were analyzed by flow cytometry. Importantly, biotin^+^ RTEs showed a bias toward the most mature M2c subset (Fig. 4 G). Thus, direct analysis of the developmental status of SP4 thymocytes undergoing PVS entry and thymic exit shows that thymic emigration is biased toward the most mature SP4 thymocytes, providing evidence for an ordered conveyor belt mechanism.

**Figure 5.**
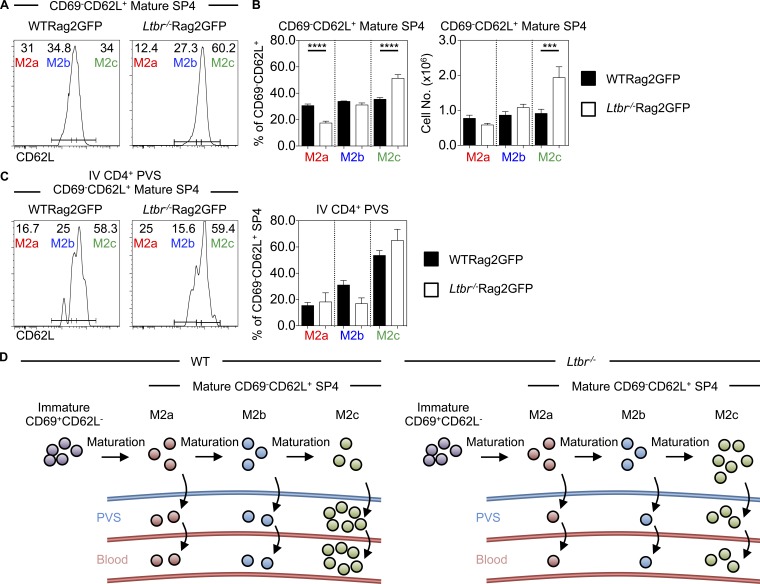
**The conveyor belt mechanism of thymocyte egress is established independently of LTβR. (A and B)** Percentage and number of M2a, M2b, and M2c subsets within CD4^+^CD8^−^TCRβ^HI^CD25^−^CD69^−^CD62L^+^Rag2GFP^+^ mature SP4 thymocytes in WTRag2GFP (black bars, *n* = 9) and *Ltbr*^−/−^Rag2GFP mice (white bars, *n* = 12). Data are pooled from five independent experiments. **(C)** M2a, M2b, and M2c anti-CD4 PE i.v. labeled mature Rag2GFP^+^ SP4 in the PVS of WTRag2GFP (left plot, black bars, *n* = 8) and *Ltbr*^−/−^Rag2GFP mice (right plot, white bars, *n* = 6). Data are pooled from three independent experiments. **(D)** A model of the relationship between LTβR and conveyor belt–driven thymic egress. A one-way ANOVA was used for all statistical analysis. All bar charts and error bars represent means ± SEM. ***, P < 0.001; ****, P < 0.0001.

Given the accumulation and prolonged medullary residency of SP4 thymocytes in LTβR-deficient mice, we examined if the requirement for LTβR during thymic emigration is explained by its role as an operator of the conveyor belt process. Thus, CD69^−^CD62L^+^ M2 SP4 in WT and *Ltbr*^−/−^ mice were subdivided into M2a, M2b, and M2c subsets on the basis of CD62L levels (Fig. 5 A). Interestingly, we found that increased numbers of CD69^−^CD62L^+^ SP4 thymocytes in *Ltbr*^−/−^ mice correlated with a specific increase in M2c cells, with M2a and M2b numbers being unaffected (Fig. 5 B). When we analyzed SP4 thymocytes in the PVS using i.v. anti-CD4 PE injection, the bias toward PE-labeled M2c cells was evident in both WT and *Ltbr*^−/−^ mice (Fig. 5 C). Thus, while LTβR quantitatively controls SP4 PVS entry (Fig. 3 E), it is not required to maintain the ordered conveyor belt nature of SP thymocyte egress via the PVS (Fig. 5 D).

In sum, we have reexamined the process of thymocyte egress and related our findings to current and opposing models. Our findings argue against the hypothesis that LTβR controls thymic egress via regulation of mTEC microenvironments. These findings parallel recent reanalysis of central tolerance, which also segregates from LTβR-dependent regulation of mTECs (Cosway et al., 2017). That LTβR-driven thymic emigration is separate from the impact of LTβR on mTEC suggests that mTEC–SP thymocyte interactions may not be essential to establish egress competency. Emigration of thymocytes from mTEC-deficient *Relb*^−/−^ thymic grafts fits with this scenario (Cowan et al., 2013). Also, as failures in medulla entry impair central tolerance (Kurobe et al., 2006), a mechanism that links thymic exit with thymocyte age may aid intrathymic retention of SP thymocytes and maximize interactions with medulla resident APCs for effective tolerance induction.

Interestingly, recent studies showed that absence of the type 2 IL4R led to PVS accumulation of SP4 thymocytes and restricted thymocyte egress (White et al., 2017). Taken together with the data presented here, this suggests that multiple pathways control stepwise thymic emigration, first from the parenchyma to the PVS (LTβR driven) and subsequently from the PVS into the blood (IL4Rα driven). Importantly, the thymic PVS acts as a site of both thymocyte egress and lymphoid progenitor entry (Mori et al., 2007). Indeed, in addition to its role in emigration, recent studies showed that LTβR controls T cell progenitor entry to the thymus (Lucas et al., 2016). While the ability of recently identified LTβR-dependent thymic portal endothelial cells (Shi et al., 2016) to control migration both into and out of the thymus is not clear, we suggest that these processes may be linked by a feedback loop involving LTβR-mediated control of endothelium and expression of LTβR ligands by SP thymocytes (Boehm et al., 2003; Shi et al., 2016). Finally, as in vivo administration of agonistic LTβR antibody was shown to enhance thymus colonization following bone marrow transplantation (Lucas et al., 2016), it will be of interest to determine any impact of anti-LTβR administration on thymic egress in the same context.

## Materials and methods

### Mice

All mice used in this study were aged 8–12 wk and on a C57BL/6 background: WT (C57BL/6), RAG-2-GFP (Rag2GFP; Yu et al., 1999), germline LTβR deficient (*Ltbr*^−/−^; Fütterer et al., 1998), *Foxn1^Cre^* (Gordon et al., 2007), *Wnt1^Cre2^* (hereafter termed *Wnt1^Cre^*; Lewis et al., 2013), *Flk1^Cre^* (Motoike et al., 2003) and *Ltbr^fl/fl^* (Wang et al., 2010). *Ltbr^fl/fl^* mice were crossed with *Foxn1^Cre^* mice to generate LTβR^TEC^ mice, *Wnt1^Cre^* mice to generate LTβR^MES^ mice, and *Flk1^Cre^* mice to generate LTβR^ENDO^ mice. In all experiments, WT C57BL/6 mice were used as controls for *Ltbr*^−/−^ mice, and *Foxn1^Cre^*, *Wnt1^Cre^*, or *Flk1^Cre^* mice were used as controls for LTβR^TEC^, LTβR^MES^, and LTβR^ENDO^ mice, respectively. WT Rag2GFP mice were crossed with *Ltbr*^−/−^ mice to generate *Ltbr*^−/−^Rag2GFP mice. Mice were housed at the University of Birmingham Biomedical Services Unit. All experimental procedures were approved by the Birmingham Animal Welfare and Ethical Review Body and were performed in accordance with UK Home Office regulations.

### Antibodies

Single-cell thymocyte and splenocyte suspensions were stained with antibodies to the following (sourced from eBioscience, unless otherwise stated): anti-CD4 Brilliant Violet (BV) 711 (RM4-5; BioLegend), CD8 BV510 (53–7.7; BioLegend), CD25 eFluor 450 (eBio3C7), CD69 PerCP-Cy5.5 (H1.2F3), CD62L APC (MEL-14; BioLegend), TCRβ APC eFluor 780 (H57.597), MHC I eFluor 450 (28–14-8), CD24 BV650 (M1/69; BD), and Foxp3 PE (FJK-16s). Intracellular staining for Foxp3 was performed using the eBioscience Foxp3/transcription factor staining buffer set according to the manufacturer’s instructions. For S1P_1_, thymocytes were first stained with purified anti-S1PR1 (mab7089; R&D Systems) and then stained with biotin anti-Rat IgG_2A_ (BioLegend), followed by Streptavidin PE Cy7 (eBioscience).

For stromal analysis, thymus samples were digested in collagenase dispase and DNase I (Sigma-Aldrich). Single-cell suspensions were stained with the following antibodies (sourced from eBioscience, unless otherwise stated): anti-CD45 APC (30-F11), EpCAM-1 PerCP eFluor 710 (G8.8), TER119 Alexa Fluor 700 (TER119), Podoplanin PE (8.1.1), Podoplanin PE Cy7 (8.1.1; BioLegend), CD31 FITC (390), LTβR Biotin (3C8), VCAM-1 Biotin (429), ICAM-1 Pacific Blue (YN1/Y.1.7.4), Ly6C PE Cy7 (HK1.4), and CD62p FITC (RB40.34; BD Bioscience). Biotin labeling was detected by staining with Streptavidin PE Cy7. Viable cells were identified using a LIVE/DEAD Fixable Dead Cell Stain Kit (Invitrogen).

### Confocal microscopy

Adult thymus tissues were snap-frozen on dry ice, mounted in Tissue-Tek optimum cutting temperature (OCT) compound (Sakura Finetek), sectioned at 7 µm thickness, and fixed in acetone. Sections were then stained using the following reagents: ERTR5 rat IgM and ERTR7 rat IgG (both gifts from E. Van Vliet, Erasmus University, Rotterdam, Netherlands), detected using anti-rat IgM Alexa Fluor 647 (Invitrogen) or anti-rat IgG Alexa Fluor 594 (Invitrogen), respectively; anti-CD31 Alexa Fluor 488 (WM-59; eBioscience) and CD8 Biotin (53–6.7; eBioscience), detected using Streptavidin Alexa Fluor 555 (Invitrogen); and DAPI (Invitrogen). Confocal microscopy of PVSs in Flk-1^cre^ and LTβR^ENDO^ sections was performed on a Zeiss Zen 880 microscope, and imaging analysis was performed using Zeiss Zen Black software. For imaging of thymus organization, a Zeiss Axio ScanZ1 was used, and images were analyzed using Zeiss Zen Blue software. To quantitate SP4 thymocytes in medullary areas, three tissue sections (cut at least 70 µm apart) were stained per thymus, and thymocytes were counted in three randomly selected 100 µm × 100 µm square medullary regions. To quantitate medullary ERTR7^+^ perivascular-associated CD31^+^ vessels, three tissue sections (cut at least 70 µm apart) were stained per thymus. The number of CD31^+^ vessels associated with ERTR7^+^ perivascular sheaths was counted in the corticomedullary region, defined as 100 µm from the CMJ.

### Measurement of proliferation by in vivo BrdU labeling

Adult mice were injected i.p. with 1.5 mg BrdU (Sigma), and tissues were harvested 18 h after injection. Thymocytes were isolated and stained for flow cytometry as outlined above. BrdU incorporation was detected after cell permeabilization using a BrdU flow kit (BD Biosciences) and staining with an Alexa Fluor 647–conjugated anti-BrdU antibody (MoBU-1; Invitrogen).

### Anti-CD4 i.v. labeling of thymocytes in the PVS

1 µg PE-conjugated anti-CD4 (GK1.5; eBioscience) was i.v. injected into mice, which were sacrificed 3 min after injection. Thymocyte suspensions were costained with anti-CD8, TCRβ, CD25, CD69, and CD62L to determine the number of anti-CD4PE i.v.^+^ cells as well as the identity of the labeled cells by flow cytometry.

### Intrathymic injection labeling with biotin

Adult mice were administered with an intrathymic injection of 10 µl of a 5 mg/ml solution of EZ-Link sulfo-NHS-LC biotin (Thermo Fisher Scientific) into each thymus lobe. Mice were harvested 18 h after injection, and thymus and spleen were stained with anti-CD4, CD8, TCRβ, CD25, CD69, and CD62L. Biotin was detected using Streptavidin-conjugated PE Cy7. Cells were then analyzed by flow cytometry.

### Quantitative PCR (qPCR)

FACS-sorted cell populations were analyzed for mRNA expression of the indicated genes by qPCR performed as described previously (Roberts et al., 2012). mRNA levels were normalized to β-actin; fold levels represent means (± SEM) of replicate reactions, and data are typical of three independently sorted biological samples. Primer sequences were as follows: β-actin QuantiTect Mm *Actb* 1SG Primer Assay (QT00095242; QIAGEN); *Spns2* forward, 5′-CCATCCTGAGTTTAGGCAACG-3′, and reverse, 5′-GATCACCTTTCTATTGAAGCGGT-3′; *Sphk1* forward, 5′-GAGCTCCGAGCTGTTTGCA-3′, and reverse, 5′-TGACACCCCCGCACGTA-3′.

### Statistical analysis

Prism 6 (GraphPad Software) was used to perform all statistical analyses. To compare multiple populations, a one-way ANOVA test was used, and an unpaired Student’s *t* test was used in all other cases. Graphs are annotated with the following indicators to signify statistical significance: *, P < 0.05; **, P < 0.01; ***, P < 0.001; and ****, P < 0.0001. Nonsignificant differences are not specified. In all figures, bar charts and error bars represent means ± SEM, respectively.

### Online supplemental material

Fig. S1 shows flow cytometric analysis of immature and mature CD4^+^8^−^ SP4 subsets using alternative phenotypic markers.

## Supplementary Material

Supplemental Materials (PDF)
