# Supplementary material for: Endothelial cells act as gatekeepers for LTβR-dependent thymocyte emigration
Source: J Exp Med. 2018 Dec 3;215(12):2984–93. doi: 10.1084/jem.20181345 (PMC6279407; doi:10.1084/jem.20181345)
Supplement: Supplemental Materials (PDF) [file JEM_20181345_sm.pdf]

Supplemental material

James et al., https://doi.org/10.1084/jem.20181345

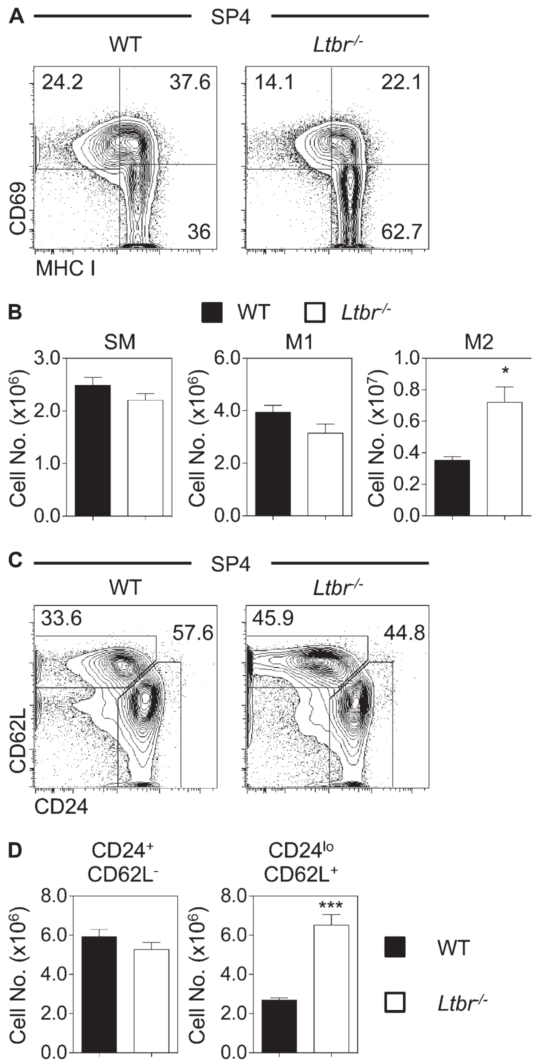

Figure S1. **Selective accumulation of mature SP4 thymocytes occurs in germline *Ltbr*$^{-/-}$ mice. (A and B)** Analysis of CD69 and MHC I expression on $CD4^{+}CD8^{-}TCR\beta^{HI}CD25^{-}$ SP4 thymocytes in WT (black) and *Ltbr*$^{-/-}$ (white) mice. **(C and D)** Analysis of CD24 and CD62L expression on SP4 thymocytes in WT (black) and *Ltbr*$^{-/-}$ (white) mice (n = 4 pooled from two independent experiments). An unpaired Student's t test was used for all statistical analysis. All bar charts and error bars represent means ± SEM. *, $P < 0.05$; ***, $P < 0.001$.
